# Analysis of Deep Drawing Process for Stainless Steel Micro-Channel Array

**DOI:** 10.3390/ma10040423

**Published:** 2017-04-18

**Authors:** Tsung-Chia Chen, Jiang-Cheng Lin, Rong-Mao Lee

**Affiliations:** Department of Mechanical Engineering, National Chin-Yi University of Technology, Taichung City 411, Taiwan; ctchen@ncut.edu.tw (T.-C.C.); nnekohiroshi01@yahoo.com.tw (J.-C.L.)

**Keywords:** micro-forming, deep drawing, micro-channel

## Abstract

The stainless steel bipolar plate has received much attention due to the cost of graphite bipolar plates. Since the micro-channel of bipolar plates plays the role of fuel flow field, electric connector and fuel sealing, an investigation of the deep drawing process for stainless steel micro-channel arrays is reported in this work. The updated Lagrangian formulation, degenerated shell finite element analysis, and the *r*-minimum rule have been employed to study the relationship between punch load and stroke, distributions of stress and strain, thickness variations and depth variations of individual micro-channel sections. A micro-channel array is practically formed, with a width and depth of a single micro-channel of 0.75 mm and 0.5 mm, respectively. Fractures were usually observed in the fillet corner of the micro-channel bottom. According to the experimental results, more attention should be devoted to the fillet dimension design of punch and die. A larger die fillet can lead to better formability and a reduction of the punch load. In addition, the micro-channel thickness and the fillet radius have to be taken into consideration at the same time. Finally, the punch load estimated by the unmodified metal forming equation is higher than that of experiments.

## 1. Introduction

Fuel cells are composed of several single-cells, which are serially connected with bipolar plates to generate sufficient current and voltage. Bipolar plates play the role of fuel input channel as well as of an electric bridge for reducing contact resistance or impedance when being connected with conducting wires [1,2,3]. Bipolar plates therefore become the key to miniaturizing the full cell, where bipolar plates represent more than 80% by weight and 40% by cost of the fuel cell [4,5]. In addition, both of the power generation efficiency and the manufacturing cost are determined by this component as well. In this case, the efficiency of heat radiating and electric conduction, which are highly related to the design of flow field, materials, and manufacturing methods of bipolar plates, should be taken into account [6,7]. Graphite and metallic materials are currently the most popular materials for bipolar plates. A composite bipolar plate made of the graphite/phenol formaldehyde resin was introduced for fuel cell applications [8]. The interaction between electrical conductivity, the shape factor and the orientation factor has been investigated. However, for reducing processing difficulty and manufacturing cost, metallic materials have received much more attention than graphite now [9,10]. The performance and long-term stability of bipolar plates made from Ti metal and stainless steels have been revealed by Park et al. [11]. The performance of Ti is lower than that of stainless steel due to the decrease in ohmic loss regions. On the other hand, stainless steels perform better as bipolar plates with regard to cell performance, cell resistance and durability. In order to produce channels for delivering fuel on metal sheets as well as reducing costs, numerous metal forming processes have been proposed such as stamping, drawing, and hydraulic pressure [10,12,13,14]. A metal bipolar plate manufactured by stamping is regarded as an alternative to the graphite bipolar plate [15]. However, the errors of micro-channel dimensions by stamping are definitely encountered, and lead to performance loss. An investigation into bipolar plate structural parameters has been carried out by Imanmehr and Pourmahmod [16]. According to the analysis results, the parameters of micro-channels have a great impact on outlet voltage at high current densities. For increasing the contact area of gas, the flow channel number should be increased as much as possible [14]. However, these micro-channels can hardly be machined by conventional methods due to their dimensions. The effect of micro-channel width on fuel cell performance has been studied [17]. The best overall performance is from the narrowest channel width, and a computational fluid dynamics model was developed to study performance variation against channel width. Similar researches regarding the effects of channel dimensions were also reported [3,13,18]. As mentioned above, the stainless steel becomes one of the most popular materials for bipolar plates due to the cost, the power generation performance, and the corrosion resistance. Though the performance of anti-corrosion for stainless steel is fine, the high strain hardening exponent of this material leads to difficulty in manufacturing. Since the micro-channel plays an important role in chemical reaction performance, such as the gas reaction area and the transport channel of solution and electron, a study regarding the deep drawing of micro-channel arrays for stainless steel sheets has been accomplished in this work. The differences between macro and micro effects are studied as well. The purpose of this work is to discuss the parameters of deep drawing process for metallic micro-channels.

## 2. Basic Theory

### 2.1. Stiffness Equation

The Lagrangian formulation can be employed to explain the properties of plastic flow. According to the Lagrangian formulation the rate equation for virtual work is as follows [19]:

(1) $$\int_{V^{E}}(\overset{\circ }{\sigma_{ij}}-2\sigma_{ik}\overset{•}{\varepsilon_{kj}})\delta \overset{•}{\varepsilon_{ij}}dV+\int_{V^{E}}\sigma_{jk}L_{ik}\delta L_{ij}dV=\int_{S_{f}}\overset{•}{f}\delta v_{i}dS$$

where *v_i_* is the velocity, the rate of nominal traction is *t_i_*, and *V* and *S_f_* are referred to the material volume and the surface respectively. Since both the virtual work rate equation and the constitutive relation are linear, they can be replaced with increments defined with respect to any monotonously increasing measures, such as the increase in the displacement of the tool. The complete global stiffness matrix can be stated as follows:

(2) [K]{Δu}={ΔF}

in which:

(3) $$[K]=\sum_{<E>}\int_{V^{E}}{\left[B\right]}^{T}([C^{ep}]-[Q])[B]dV+\sum_{<E>}\int_{V^{E}}{\left[E\right]}^{T}[Z][E]dV$$

The term {Δu} in Equation (2) is the increment of nodal displacement and the increase of nodal force is {ΔF}. [*K*] and [*C^ep^*] indicate the global tangent stiffness matrix and the elemental elastic-plastic constitutive matrix, respectively. The strain rate velocity matrix and the velocity gradient matrix are [*B*] and [*E*] respectively. [*Q*] and [*Z*] correspond to the stress correction matrices against each deformation stage respectively.

### 2.2. Selective Reduced Integration Formulation

The plastic medium volume is incompressible. Therefore, over-strong constraint for thin plates will be engaged when the full integration technique is applied for finite elements. This situation is due to the setting of no shear strain γ_xz_ and γ_yz_ during the deformation [20]. For solving such problems, in which the volumetrically in stiff contribution is involved, the selective reduced integration has been verified as an effective method [21]. The generalized formulation of selective reduced integration proposed by Hughes [22] has been employed in this work. In addition, the four-node quadrilateral degenerated shell element [23] is utilized in the intensive analysis.

### 2.3. Scale Factors for Micro-Forming Process of Sheet Metal

Generally, the size effect can be neglected for the thickness of sheet metal within 1.0 mm. However, the metallic sheet thickness in micro-forming process is of micron range and the traditional material model is therefore no longer suitable for analyses under this condition. A modified model without considering size effect has been employed in this work. A traditional material model can be defined as follows:

(4) $$\bar{\sigma }=K{\left(\varepsilon_{0}+\bar{\varepsilon_{p}}\right)}^{n}$$

The thickness of the metallic sheet in the following analysis is 50 μm. As a result, the size effect should be taken into account for the amendment of stress-strain relations. Consequently, Equation (4) is further amended as follows:

(5) $$\bar{\sigma }(t,\bar{\varepsilon })=aKe^{bt}{\left(\varepsilon_{0}+\bar{\varepsilon_{p}}\right)}^{n(ce^{dt}-1)}$$

where *a*, *b*, *c*, *d* are the correction coefficients. *t* is the metallic sheet thickness. These correction coefficients can be given according to Ref. [24] and Equation (5) therefore becomes

(6) $$\bar{\sigma }(t,\bar{\varepsilon })=0.73667Ke^{0.3152t}{\left(\varepsilon_{0}+\bar{\varepsilon_{p}}\right)}^{n(1.0106e^{-0.01029t}-1)}$$

Both the traditional material model, Equation (4), and the modified material model, Equation (6), are enclosed in the following finite element analyses. Experiments are conducted for the performance verification of these two models.

## 3. Numerical Analysis for Micro-Channel Array Forming Process

Four-node quadrilateral degenerated shell elements are utilized in this work for deriving the stiffness matrix, and Finite Element Analysis (FEA) [25] is employed for the pre-processing and post-processing. Since the die and stainless steel plates are supposed to be symmetric, the simulation is performed against a 1/4 figure to effectively reduce the analysis time for arithmetic processing. The die and the metallic plate are then meshed with quadrilateral elements. These meshed data are transformed to the 3D elastic-plastic FEA program for the following simulation analysis.

**Figure 1:**
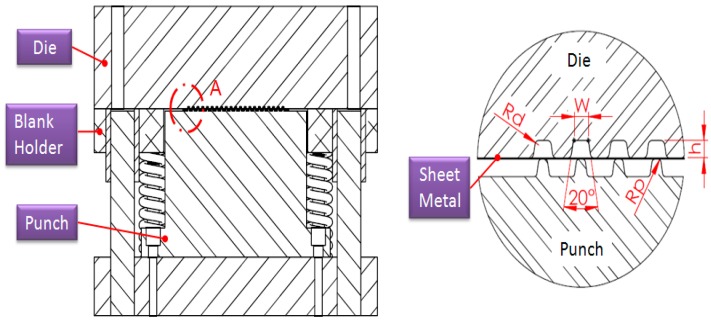
Micro-channel array forming process for stainless steel plates.

**Figure 2:**
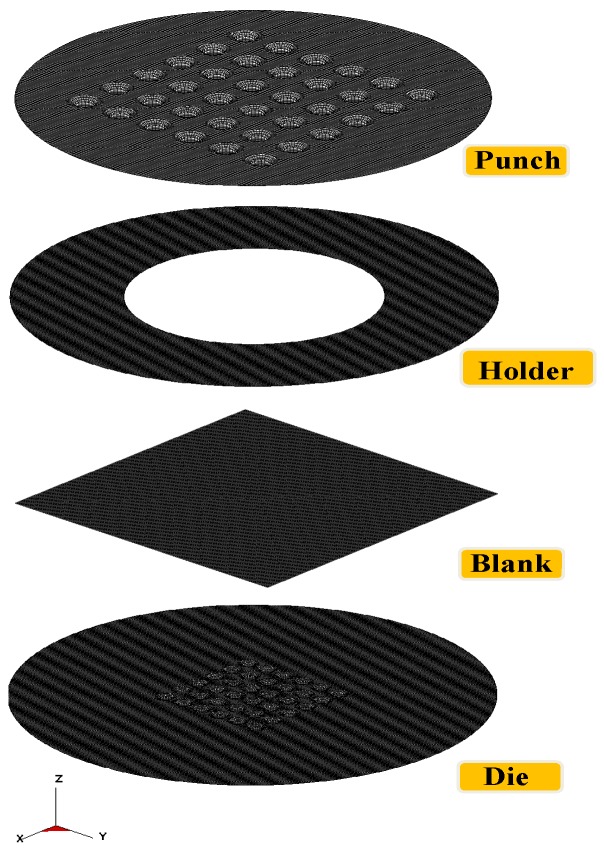
Finite element model.

**Figure 3:**
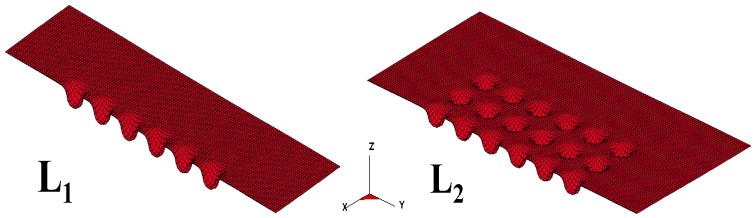
Measurement schematic diagram.

**Figure 4:**
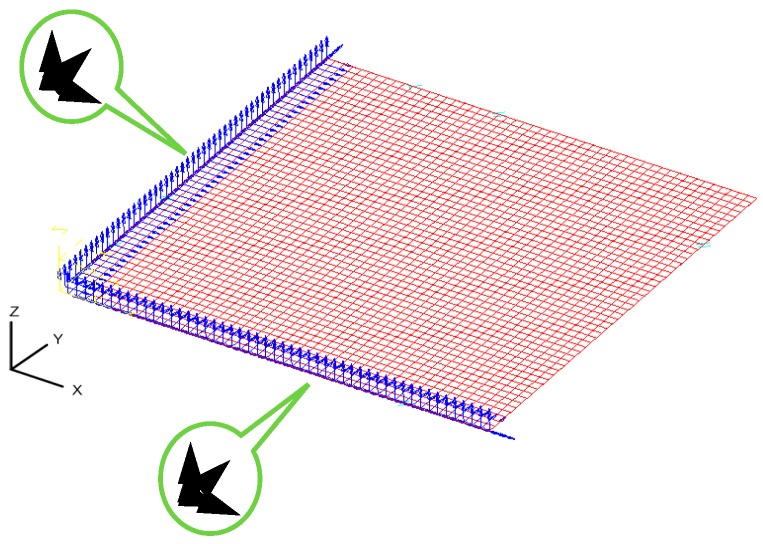
Boundary conditions of blank.

Since the die is too small to be manufactured by conventional methods, electric discharge machining is utilized for the die fabrication. The micro-channel array forming process is illustrated in Figure 1. The dimensions of tools for the micro-channel array forming process are listed in Table 1, where the die gap is set to be 1.1 times the plate thickness, in accordance with traditional empirical rules. Since the thickness of the stainless steel sheet used in this study is 0.05 mm, the die gap is designed to be 0.055 mm. The mechanical properties of the stainless steel sheet is illustrated in Table 2. The die and the metallic plate are meshed into quadrilateral elements (see Figure 2). These meshed data are then loaded into the 3D elastic-plastic FEA program for the following numerical analysis. Figure 3 is the cross-section schematic diagram of the processed plate. L_1_ and L_2_ sections are the main objects for the following investigation, including the thickness distribution and the contour. In the micro-channel array deep drawing process, the die will directly contact the blank surface. That means the node for die contact and separation should be defined. Nodes are therefore divided into two categries: contact node and free node. The boundary conditions of the material meshing quadrilateral elements are demostrated in Figure 4.

## 4. Experimental Results and Discussion

**Figure 5:**
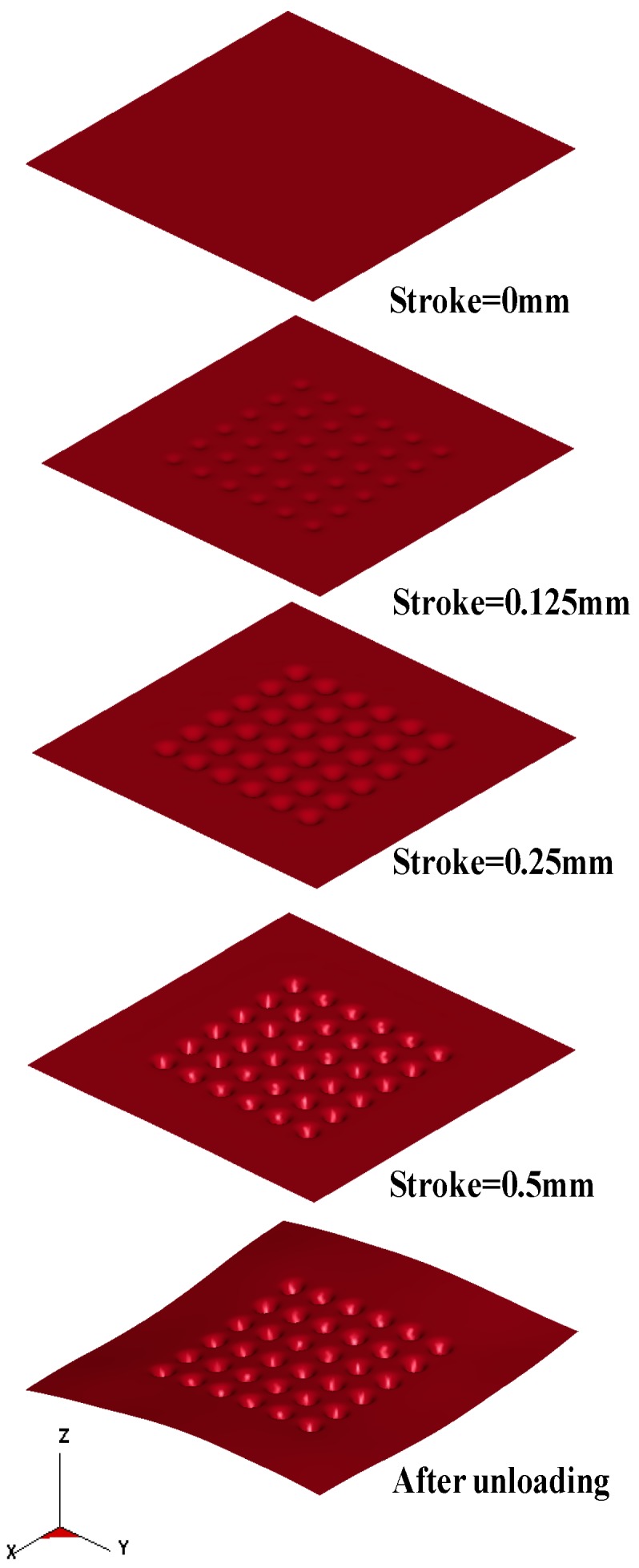
The geometric deformation of thin metal sheet against various strokes.

**Figure 6:**
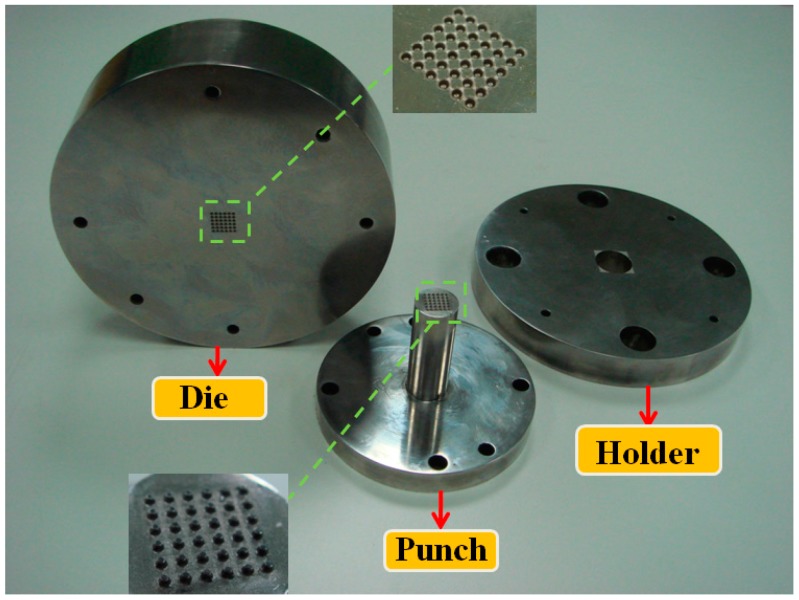
The prototype of tools for the micro-channel array forming process.

**Figure 7:**
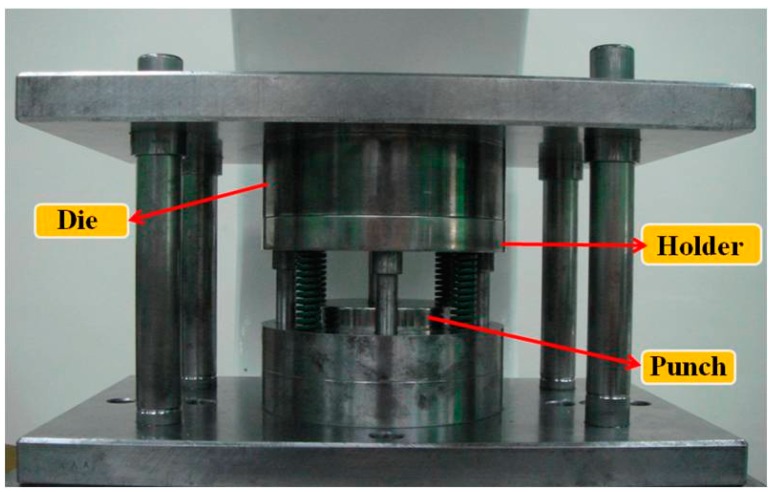
The test rig for the micro-channel array forming process.

**Figure 8:**
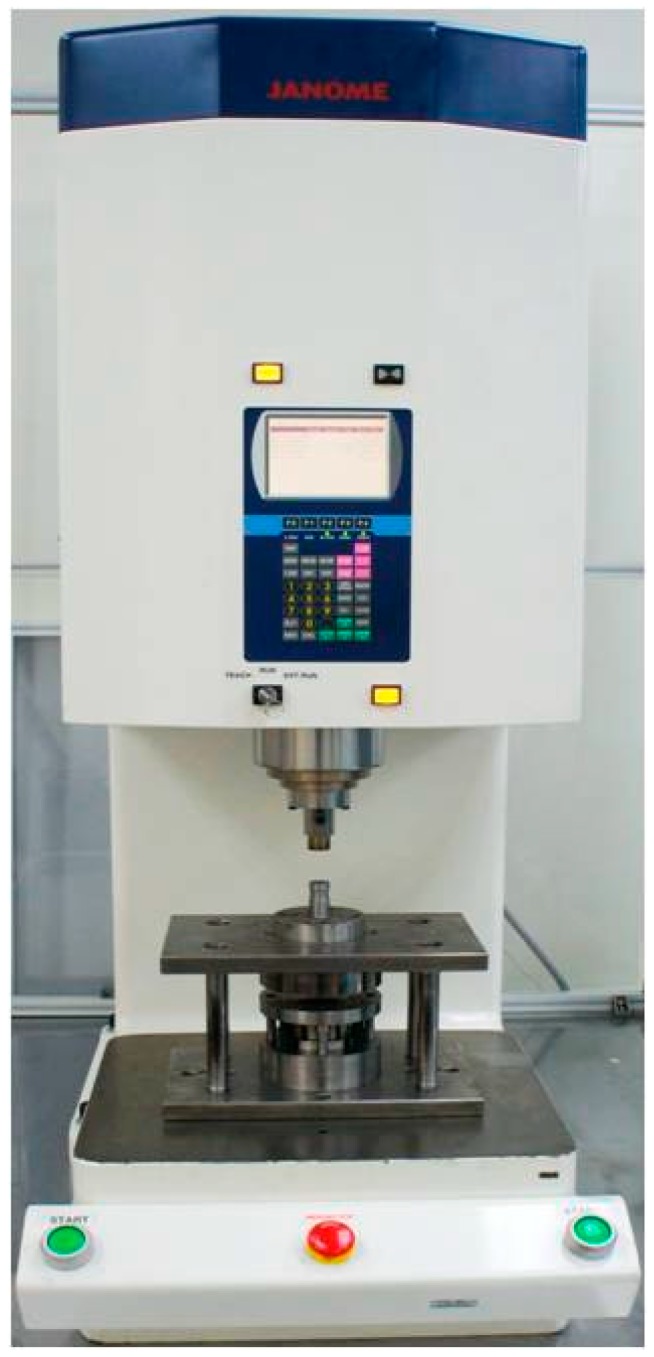
The electro press for the micro-channel array forming process (JANOME JP-5004).

The micro-channel array deep drawing process for stainless steel plate is shown in Figure 5. In total, there are five steps in this geometric deformation process. The plate is gradually deformed during micro-channel forming until the unloading state. Throughout the entire micro-channel forming process, the contact, separation, and friction conditions can be accurately estimated by the *r*-minimum (*r_min_*) rule. The *r_min_* rule is mainly for the definition of boundary conditions for degenerated four-node shell elements during deformation analyses. Figure 6 is the prototype of tools employed in the micro-channel array drawing experiment, including punch, die bottom, and blank holder. The test rig and the electro press (JANOME JP-5004, JANOME Sewing Machines Co., Ltd., Tokyo, Japan) for micro-channel array forming process are shown in Figure 7 and Figure 8 respectively.

### 4.1. Comparisons between Experimental Results and Simulation Results

**Figure 9:**
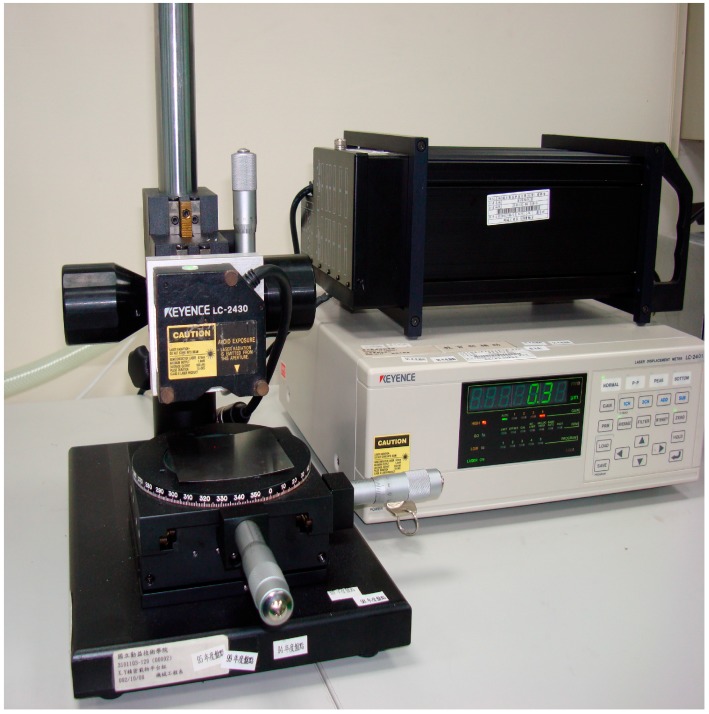
Laser displacement system (Keyence LC-2430) for contour measurements of stamped stainless steel plates.

**Figure 10:**
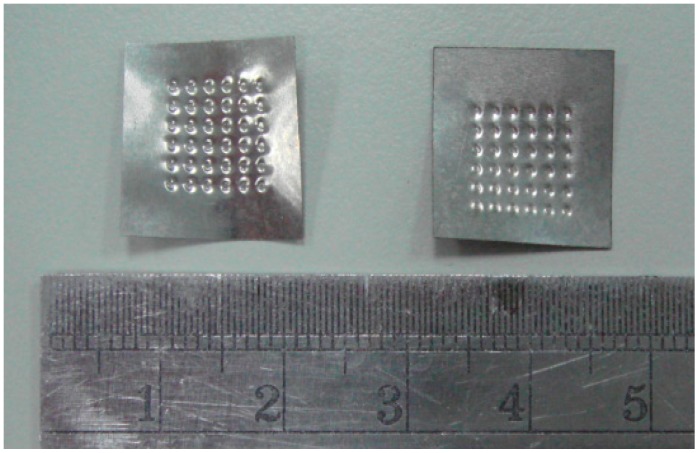
Processed workpiece of micro-channel array forming process.

Two models, i.e., macro and micro, are employed in the simulation of the micro-channel array deep drawing process for stainless steel plate. Since the thickness of the stainless steel plate for experiments is merely 50 μm, the contour measurements of the finished workpieces is accomplished by using a laser displacement system, as shown in Figure 9 (Keyence LC-2430, Osaka, Japan). Some of the processed workpieces are shown in Figure 10.

**Figure 11:**
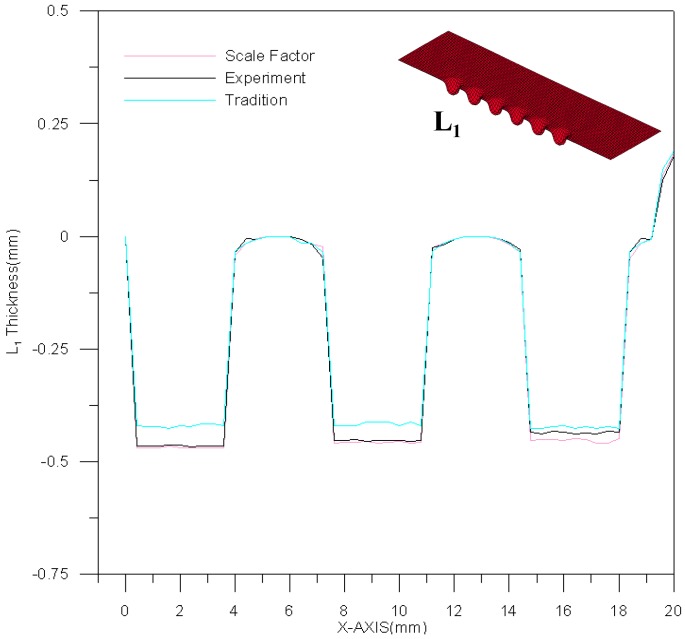
The contour comparison of L_1_ section.

**Figure 12:**
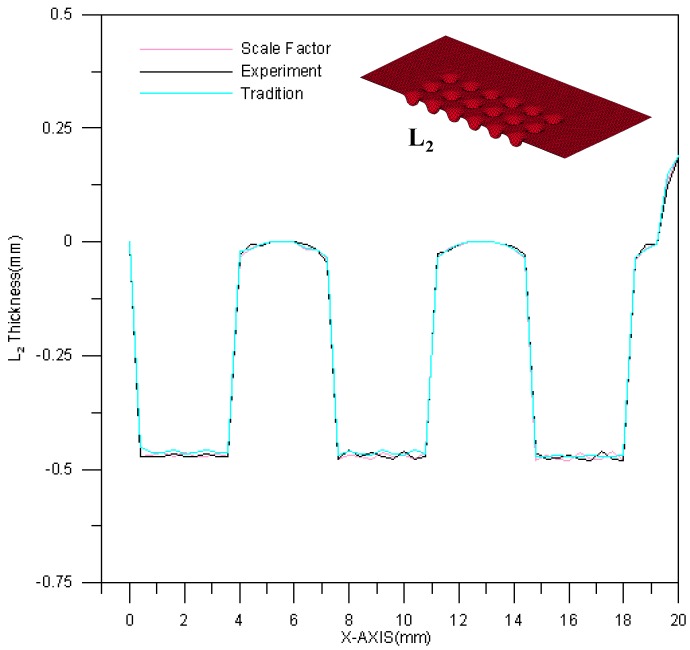
The contour comparison of L_2_ section.

Figure 11 is a comparison between theoretical estimations and experimental results for the L_1_ section contour. The channel depth is about 0.5 mm. However, the effect of material springback results in an upward curl of the peripheral part of the stainless steel plate. Namely, the channel will deviate from the center of stainless steel plate and move downward. As a result, the deformation trend could be predicted more precisely by the material model of modified scale factor than by traditional material models. A comparison between theoretical estimations and experimental results for the L_2_ section contour is shown in Figure 12. Similarly, the difference in channel depth between these three results (experimental results, traditional material model and modified scale-factor material model) is mainly due to the peripheral curvature of the stainless steel plate. The maximum depth error between these two material models is about 0.035 mm. The performance of the modified scale factor material model is still better than that of traditional material model for L_2_ section contour estimation.

**Figure 13:**
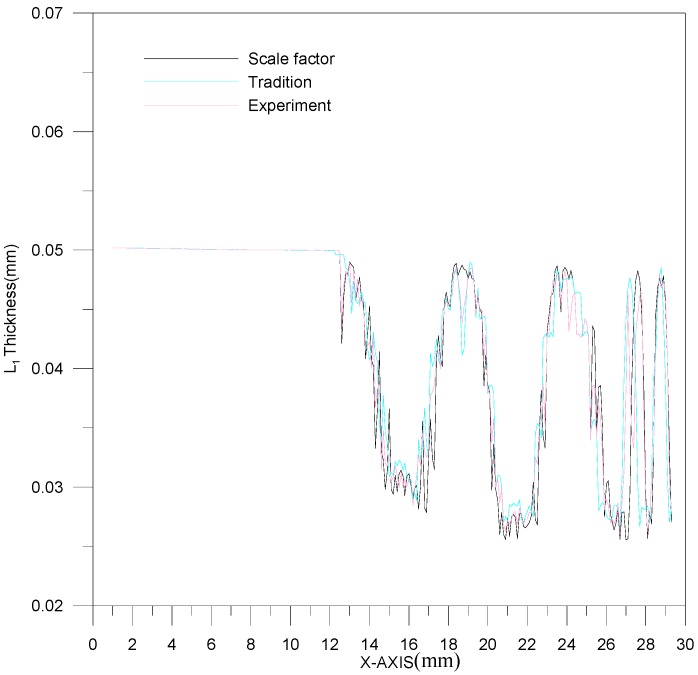
The thickness variation of the L_1_ section.

**Figure 14:**
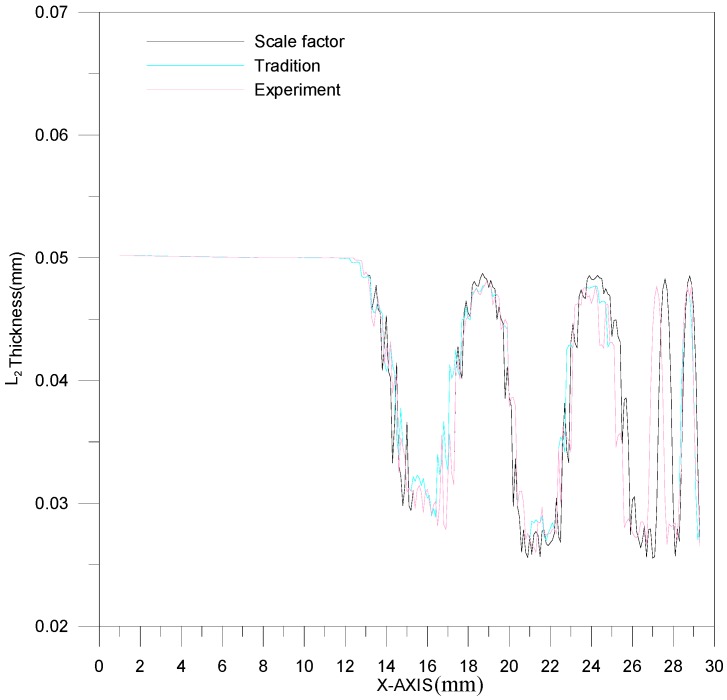
The thickness variation of the L_2_ section.

The thickness of the L_1_ section is shown in Figure 13. The range of thickness variation is 0.0264~0.033 mm. Based on Figure 13, the experimental result is between the results of the two theoretical models. In addition, among all the processed channels in one plate, the first channel is the thinnest. The thickness variations for these three results (experimental results, traditional material models and modified scale-factor material model) are similar, and no significant error is observed. The thickness of the L_2_ section is shown in Figure 14. The L_2_ section is thicker than the L_1_ section. The thickness difference is about 0.003 mm.

**Figure 15:**
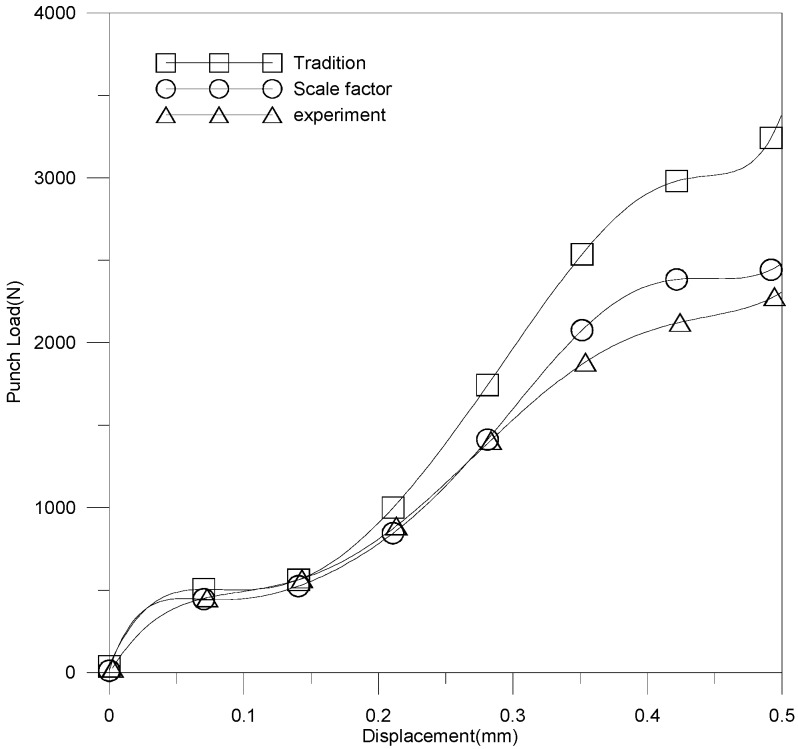
The relationship of punch load and stroke.

The relationship of punch load and stroke is revealed in Figure 15. The curve variations for these three results (experimental results, traditional material model and modified scale-factor material model) are similar; namely, the punch load increased with increasing stroke. However, significant errors are observed once the stroke exceeds a threshold. The large error is due to the increase in contact area between die and blank. According to Figure 15, the simulation results for the modified scale-factor material model is much better than those of traditional material model.

### 4.2. Effect of Tool Radius on the Micro-Channel Array Forming Process of Stainless Steel Plates

**Figure 16:**
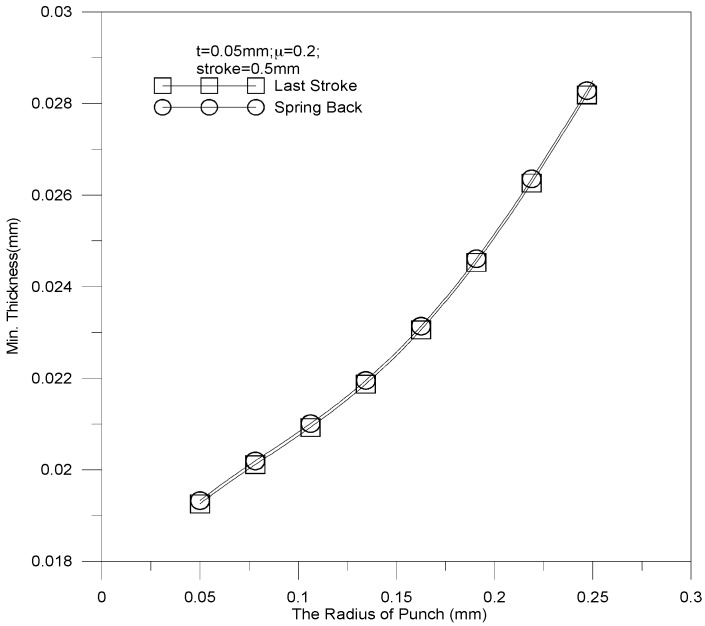
The relationship between sheet thickness and punch fillet radius.

The dimensional effect of punch fillet radius on the thickness of stainless steel plate is demonstrated in Figure 16. The parameters of the micro-channel array forming process are as follows:

* Punch fillet radius: 0.05 mm, 0.1 mm, 0.15 mm, 0.2 mm, and 0.25 mm
* Stroke: 0.5 mm
* Coefficient of friction: 0.2
* Blank thickness: 0.5 mm

**Figure 17:**
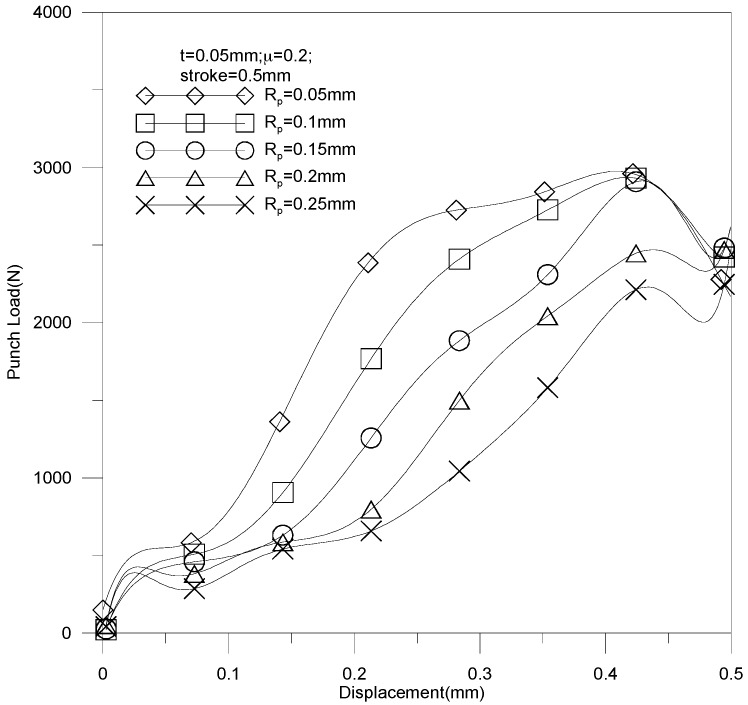
Punch load against stroke under various punch fillet radiuses.

The thickness increases linearly when the fillet radius is increased. The minimal thickness is 0.0201 mm against a punch fillet radius of 0.05 mm. The thickest sheet thickness is 0.0278 mm against a punch fillet radius of 0.25 mm. Apparently, an acute angle of channel would be formed more easily by a small punch fillet radius than by a large one, and may lead to material fracture. The punch load against stroke under various punch fillet radiuses is reported in Figure 17. It should be noticed that a small punch fillet radius leads to a large punch load; e.g., the maximal load (about 5000 N) is induced by a tool radius of 0.05 mm. As a result, the punch load can be reduced by increasing the punch fillet radius.

**Figure 18:**
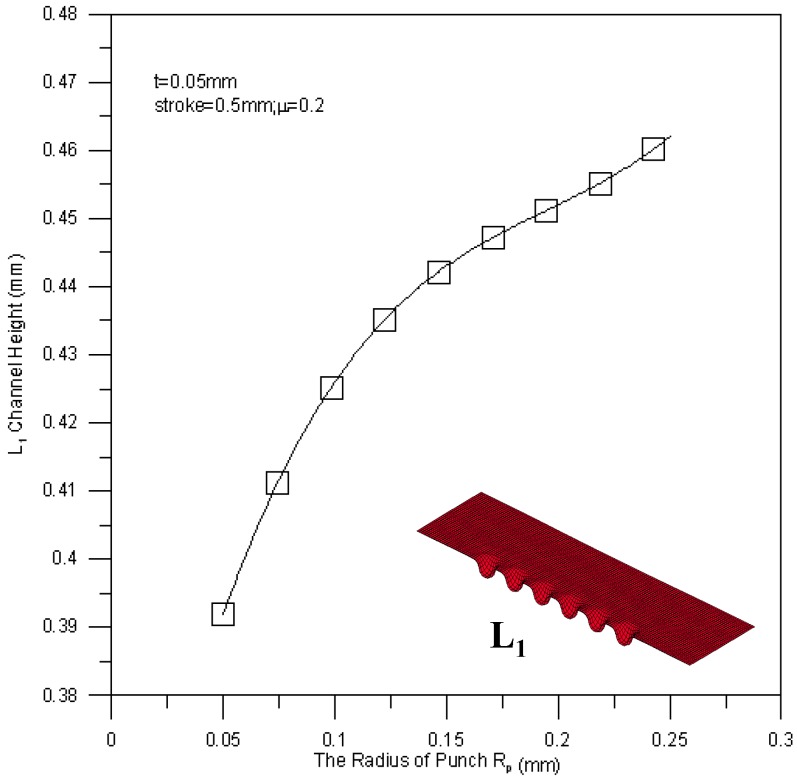
The relationship between the tool radius and the channel height of the L_1_ section.

**Figure 19:**
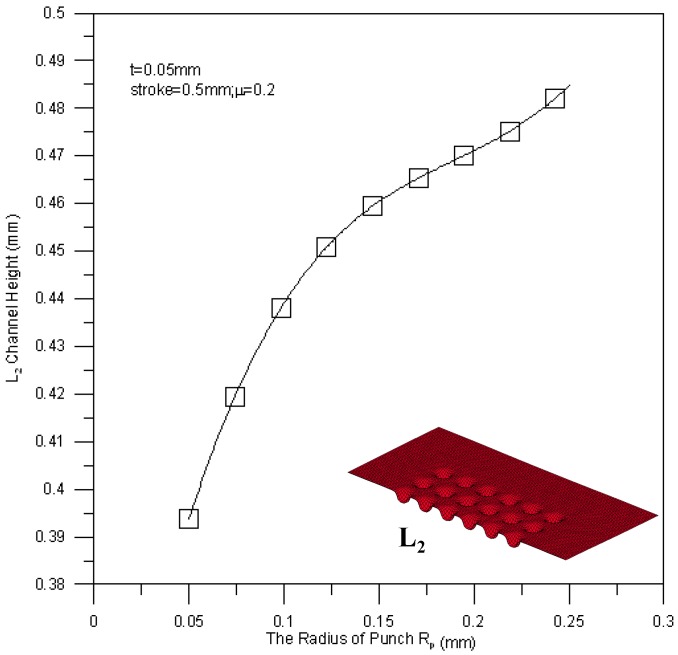
The relationship between the tool radius and the channel height of the L_2_ section.

Figure 18 shows the relationship between the mean channel height of the L_1_ section and the punch fillet radius. A large punch fillet radius leads to a high channel height of the L_1_ section, e.g., the maximum mean channel height is achieved by a punch fillet radius of 0.25 mm. Since the flowability of material is regulated by the fillet radius, the deep drawing process can be performed more smoothly when a large fillet radius is applied. The mean channel height of the L_2_ section against fillet radius is shown in Figure 19. The variation trend of the L_2_ section height is similar to that of the L_1_ section height, but the measured height of the L_2_ section is a little bit higher than the height of the L_1_ section (about 0.02 mm). This is due to the reducing of the peripheral contact area of the channel by buckling.

**Figure 20:**
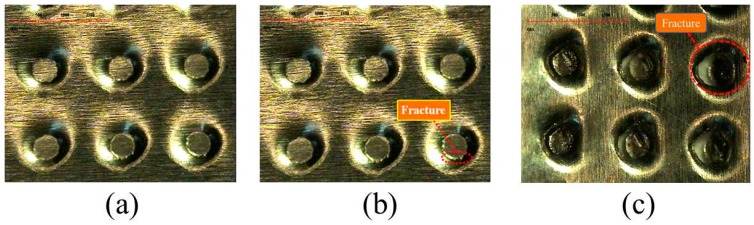
Processed stainless steel plate (**a**) stroke of 0.5 mm; (**b**) stroke of 0.55 mm; (**c**) stroke of 0.6 mm.

The practically processed workpieces are shown in Figure 20. In total, five kinds of strokes have been applied in the experiments, including 0.4 mm, 0.45 mm, 0.5 mm, 0.55 mm and 0.6 mm. Significant fractures have been observed in the plates at strokes of 0.55 mm and 0.6 mm. As a result, the stroke limitation for micro-channel array deep drawing processes in this work is about 0.5 mm.

## 5. Conclusions

3D elastic-plastic finite element analysis has been employed in this work to analyze the micro-channel array deep drawing process for stainless steel plate. The *r*-minimum rule is applied to simplify the calculation during the non-linear process. The main results are summarized as follows:

(1) The maximum channel depth for a 50 μm stainless steel sheet is about 0.5 mm. This is verified by both of the simulation results and the experimental results.
(2) On the basis of the finished workpieces, the material fracture is mainly located in the contact area between the punch and the bottom die fillet. As a result, more attention should be devoted to fillet radius design.
(3) The deep drawing process can be performed more smoothly when a large fillet radius is applied. In other words, a small tool fillet radius may lead to an increase of fracture.
(4) The simulation performance of the modified scale-factor material model is better than that of the traditional material model.

## Figures and Tables

**Table 1:** Dimensions of tools.

Items	Dimensions (mm)
Width of channel (*W*)	0.75
Height of channel (*h*)	0.5
Radius of die (*R_d_*)	0.25
Radius of punch (*R_p_*)	0.2

**Table 2:** Mechanical properties of stainless steel SUS304.

Material (SUS304)	*E* (GPa)	ν	σ_y_ (MPa)	*K* (MPa)	*n*	ε_0_
Tradition	207	0.3	341	1819	0.576	0.077
Scale Factor	198	0.3	306	1361	0.582	0.077

Remarks: *E*: Modulus of elasticity; ν: Poisson’s ratio; σ_y_: Yield stress.
